# The Leukodystrophy Spectrum in Saudi Arabia: Epidemiological, Clinical, Radiological, and Genetic Data

**DOI:** 10.3389/fped.2021.633385

**Published:** 2021-05-13

**Authors:** Majid Alfadhel, Mohammed Almuqbil, Fuad Al Mutairi, Muhammad Umair, Mohammed Almannai, Malak Alghamdi, Hamad Althiyab, Rayyan Albarakati, Fahad A. Bashiri, Walaa Alshuaibi, Duaa Ba-Armah, Mohammed A. Saleh, Ali Al-Asmari, Eissa Faqeih, Waleed Altuwaijri, Ahmed Al-Rumayyan, Mohammed Ali Balwi, Faroug Ababneh, Abdulrahman Faiz Alswaid, Wafaa M. Eyaid, Naif A. M. Almontashiri, Amal Alhashem, Khalid Hundallah, Aida Bertoli-Avella, Peter Bauer, Christian Beetz, Muhammad Talal Alrifai, Ahmed Alfares, Brahim Tabarki

**Affiliations:** ^1^Division of Medical Genetics, Department of Pediatrics, King Abdulaziz Medical City, Ministry of National Guard Health Affairs (MNG-HA), Riyadh, Saudi Arabia; ^2^Medical Genomics Research Department, King Abdullah International Medical Research Center (KAIMRC), King Saud Bin Abdulaziz University for Health Sciences, Ministry of National Guard Health Affairs (MNGH), Riyadh, Saudi Arabia; ^3^College of Medicine, King Saud bin Abdulaziz University for Health Sciences, King Abdulaziz Medical City, Ministry of National Guard Health Affairs (MNG-HA), Riyadh, Saudi Arabia; ^4^Division of Neurology, Department of Pediatrics, King Abdullah Specialist Children's Hospital, King Abdulaziz Medical City, Ministry of National Guard Health Affairs (MNG-HA), Riyadh, Saudi Arabia; ^5^Section of Medical Genetics, Children's Specialist Hospital, King Fahad Medical City, Riyadh, Saudi Arabia; ^6^Medical Genetics Division, Department of Pediatrics, College of Medicine, King Saud University, Riyadh, Saudi Arabia; ^7^Division of Neurology, Department of Pediatrics, College of Medicine, King Saud University, Riyadh, Saudi Arabia; ^8^Pathology and Laboratory Medicine, King Abdulaziz Medical City, Riyadh, Saudi Arabia; ^9^Center for Genetics and Inherited Diseases, Taibah University, Almadinah Almunwarah, Saudi Arabia; ^10^Faculty of Applied Medical Sciences, Taibah University, Almadinah Almunwarah, Saudi Arabia; ^11^Division of Genetics, Department of Pediatrics, Prince Sultan Military Medical City, Riyadh, Saudi Arabia; ^12^Department of Anatomy and Cell Biology, College of Medicine, Alfaisal University, Riyadh, Saudi Arabia; ^13^Division of Neurology, Department of Pediatrics, Prince Sultan Military Medical City, Riyadh, Saudi Arabia; ^14^CENTOGENE AG, Rostock, Germany; ^15^Department of Pediatrics, Almulyda, Saudi Arabia. King Abdullah International Medical Research Center (KAIMRC), Qassim University, Riyadh, Saudi Arabia

**Keywords:** leukodystrophy, Saudi Arabia, neurometabolic, metachromatic leukodystrophy, novel mutations

## Abstract

**Background:** Leukodystrophies (LDs) are inherited heterogeneous conditions that affect the central nervous system with or without peripheral nerve involvement. They are individually rare, but collectively, they are common. Thirty disorders were included by the Global Leukodystrophy Initiative Consortium (GLIA) as LDs.

**Methods:** We conducted a retrospective chart review of a consecutive series of patients diagnosed with different types of LD from four large tertiary referral centers in Riyadh, Saudi Arabia. Only those 30 disorders defined by GLIA as LDs were included.

**Results:** In total, 83 children from 61 families were identified and recruited for this study. The male-to-female ratio was 1.5:1, and a consanguinity rate of 58.5% was observed. An estimated prevalence of 1:48,780 or 2.05/100,000 was observed based on the clinical cohort, whereas a minimum of 1:32,857 or 3.04/100,000 was observed based on the local genetic database. The central region of the country exhibited the highest prevalence of LDs (48.5%). The most common LD was metachromatic leukodystrophy (MLD), and it accounted for 25.3%. The most common disorder based on carrier frequency was AGS. Novel variants were discovered in 51% of the cases, but 49% possessed previously reported variants. Missense variants were high in number and accounted for 73% of all cases. Compared with other disorders, MLD due to saposin b deficiency was more common than expected, Pelizaeus-Merzbacher-like disease was more prevalent than Pelizaeus-Merzbacher disease, and X-linked adrenoleukodystrophy was less common than expected. The mortality rate among our patients with LD was 24%.

**Conclusion:** To the best of our knowledge, this is the largest cohort of patients with LD from Saudi Arabia. We present epidemiological, clinical, radiological, and genetic data. Furthermore, we report 18 variants that have not been reported previously. These findings are of great clinical and molecular utility for diagnosing and managing patients with LD.

## Introduction

Leukodystrophies (LDs) are inherited heterogeneous conditions affecting the central nervous system with or without peripheral nerve involvement (1, 2). They are individually rare but are collectively common. Their prevalence is not yet known because of challenges in diagnosis; however, it is estimated to be in the range of 1:7663–1:50,000 (1). The exact definition of LD is not well-described and is further complicated by the existence of other LD terms used in the literature (2). In addition to primary hereditary LD, this condition can be secondary to vascular, infectious, toxic, and other insults to the white matter. In 2015, 13 experts representing the Global Leukodystrophy Initiative Consortium (GLIA) used a modified Delphi-based approach to achieve a case definition of LDs. Thirty disorders were included in this category (Table 1) (2).

**Table 1:** Thirty disorders classified as leukodystrophy by Global Leukodystrophy Initiative Consortium (GLIA).

**#**	**Disorder**
1	Pol-III related disorders [4H syndrome (hypomyelination, hypodontia and hypogonadotropic hypogonadism)]
2	18q minus syndrome
3	X linked Adrenoleukodystrophy (X-ALD)
4	Adult onset leukodystrophy with neuroaxonal spheroids and pigmented glia (including hereditary)
5	Diffuse leukoencephalopathy with spheroids, HDLS, and Pigmentary type of orthochromatic leukodystrophy with pigmented glia, POLD)
6	Aicardi-Goutières Syndrome (AGS)
7	Alexander Disease (AxD)
8	Autosomal Dominant Leukodystrophy with Autonomic disease (ADLD)
9	Canavan disease
10	Cerebrotendinous Xanthomatosis (CTX)
11	Chloride Ion Channel 2 (ClC-2) related leukoencephalopathy with intramyelinic edema
12	eIF2B related disorder [Vanishing White Matter Disease or Childhood ataxia with central nervous system hypomyelination (CACH)]
13	Fucosidosis
14	Globoid cell Leukodystrophy (Krabbe)
15	Hypomyelination with atrophy of the basal ganglia and cerebellum (H-ABC)
16	Hypomyelination with Brainstem and Spinal Cord involvement and Leg Spasticity (HBSL)
17	Hypomyelination with congenital cataract (HCC)
18	Leukoencephalopathy with brainstem and spinal cord involvement and lactate elevation (LBSL)
19	Leukoencephalopathy with thalamus and brainstem involvement and high lactate (LTBL)
20	Megalencephalic Leukoencephalopathy with subcortical cysts (MLC)
21	Metachromatic Leukodystrophy (MLD) and its biochemical variants
22	Oculodentodigital dysplasia
23	Pelizaeus Merzbacher disease (PMD)
24	Pelizaeus Merzbacher like-disease (PMLD)
25	Peroxisomal Biogenesis disorders (including Zelleweger, neonatal Adrenoleukodystrophy and Infantile Refsum)
26	Polyglucosan Body Disease (PGBD)
27	RNAse T2 deficient leukoencephalopathy
28	Sialic acid storage disorders (Salla disease, Infantile Sialic Acid Storage Disease and Intermediate form)
29	Single enzyme deficiencies of peroxisomal fatty acid beta oxidation (including only D-Bifunctional Protein Deficiency; Sterol Carrier Protein X (SCPx) deficiency; Peroxisomal acyl-CoA-Oxidase Deficiency)
30	Sjögren-Larsson syndrome
	SOX10-associated PCWH: peripheral demyelinating neuropathy, central dysmyelinating leukodystrophy, Waardenburg syndrome, and Hirschsprung disease

The clinical features of LD can present at or shortly after birth for some types of LDs, whereas others develop later in childhood, adolescence, or even during adulthood. All LDs share selective white matter abnormalities upon neuroradiological imaging of the brain. White matter deterioration occurs concurrently with clinical neuroregression of the associated skills. Several LDs are degenerative; however, some are limited to white matter function impairment. The clinical course is heterogeneous in nature; some patients experience progressive disease, and others may have static disease or exhibit improvement over time. The typical clinical presentation in childhood is neurological and mainly affects motor functions. This is dissimilar to primary neuronal disorders that present initially with cognitive defects and epilepsy (3, 4). The common scenarios include but are not limited to neuroregression in a proband after normal development with loss of milestones and hypotonia that progresses to peripheral spasticity, optic atrophy, and global developmental delay. The initial symptoms in older children can manifest as frequent falls or a clumsy gait. Adult-onset LD is largely similar to child-onset LD with typical progressive cognitive or neuropsychiatric difficulties. Cerebral defects are characterized by changes in personality, emotional disturbances, impaired attention, and memory loss. Several LDs possess specific clinical features, such as X-linked adrenoleukodystrophy (X-ALD), that is characterized by adrenal insufficiency, patients possessing metachromatic leukodystrophy (MLD), Krabbe disease that is characterized by peripheral neuropathy, and macrocephaly that occurs in Canavan and Alexander diseases. Patients with 4H syndrome suffer from hypogonadotropic hypogonadism. Optic atrophy is notable in Canavan disease and vanishing white matter disease, whereas brain calcification is common in Aicardi-Goutieres syndrome (AGS) (3, 4).

In this study, we report the largest cohort of patients with LD in Saudi Arabia. We estimated the prevalence, common LD types, and radiological and molecular data associated with LD.

## Methods

### Ethical Approval

The study was approved by the Institutional Review Board of King Abdullah International Medical Research Centre (KAIMRC), Riyadh (Ref. RC19/120/R). Written informed consent for the publication of related data was obtained from the parents of the enrolled patients.

### Patients

This is a retrospective chart review study of a consecutive series of patients diagnosed with different types of LD from four large tertiary referral centers in Riyadh, Saudi Arabia (January 2015 to January 2020). Patient records were reviewed to collect data on variables that included clinical diagnoses, number of patients, sex, age of onset, age at diagnosis, current age, neuroregression, global developmental delay, seizure and its types, magnetic resonance imaging (MRI) findings, long-term outcomes, and genetic results. The standard threshold criteria were applied to diagnose LD, and these included neurological clinical presentation in addition to white matter abnormalities in MRI of the brain. These diagnoses were also confirmed using DNA molecular genetic testing.

### Genetic Testing

The diagnosis of LD was confirmed by whole exome sequencing (WES) or whole genome sequencing (WGS) of genomic DNA for the majority of patients, but a small number of patients were diagnosed according to relevant gene panels or single gene testing. All molecular genetic studies were performed by accredited commercial laboratories, such as CENTOGENE (https://www.centogene.com/). Cases with negative gene panels were reflexed to WES/WGS as described elsewhere (5, 6). The variants were classified according to the guidelines of the ACMG (PMID: 25741868). A minor allele frequency of <0.001 was selected based on our internal database (1,410 exomes), and 13,180 WES/WGS were performed at CENTOGENE (https://www.centogene.com/), the Genome Aggregation Database (gnomAD), and Exome Aggregation Consortium (ExAC) databases. Additionally, the variants should be fully segregated within available family members. Variants obtained after WES/WGS were analyzed and filtered using standard methods (Supplementary Figure 1). All discovered variants were confirmed by bidirectional Sanger sequencing following standard methods. For missense variants, *in silico* analysis tools, including Polyphen2 (http://www.genetics.bwh.harvard.edu/pph2/), SIFT (http://sift.bii.a-star.edu.sg/), Varsome (https://varsome.com/), and MutationTaster (http://www.mutationtaster.org/), were used to predict the pathogenicity of the variants. For splice site variants, Human Splicing Finder (HSF; http://www.umd.be/HSF3/index.html) was used as a prediction tool.

## Results

### Cohort Description

A total of 83 children from 61 families were identified and recruited for this retrospective study. The consanguinity rate among the study participants was 58.5%. The age of LD onset ranged from 0 to 7 years, age at diagnosis ranged from 0 to 19 years, and age at follow-up ranged from 0.3 to 25 years. Tables 2, 3 summarize the clinical and genetic information for all of the cases. The estimated prevalence of LD was 1:48,780 or 2.05/100,000. Upon reviewing our in-house genome database of 1,410 individuals, the estimated prevalence was calculated to be in the range of 1:32,857 to 3.04/100,000. Common clinical features included global developmental delay (97.5%), neuroregression (40%), and seizure (50%). The types of seizures included generalized tonic-clonic, focal colonic, myoclonic, and infantile spasms.

**Table 2:** Summary of demographic and clinical data.

**#**	**Disorder/Total**	**Gene**	**Number of patients**	**M:F**	**Age of onset (yrs)**	**Age of diagnosis (yrs)**	**Current age (years)**	**Neuroregression**	**Global developmental delay**	**Seizure**	**Type of seizure**	**MRI findings**	**Long term outcome**
1	Metachromatic leukodystrophy (MLD) (21)	ARSA	11	5:6	0.8-1.5	2–4	6–11	11/11	11/11	6/11	GTC, IS	Supratentorial deep periventricular white matter abnormal signal with the sparing of the subcortical U fibers.	7 deceased and 4 alive with severe GDD
		PSAP	10	8:2	1.5–3	2.5–8	5–12	09/10	10/10	2/10	GTC	Diffuse bilateral cerebral subcortical and deep white matter T2/FLAIR hyperintensity sparing the subcortical U-fibers, involving the posterior limbs of the internal capsules and middle cerebellar peduncles.	Alive with severe GDD
2	Leukoencephalopathy with vanishing white matter (11) (VWM)	EIF2B4	7	5:2	0.9–2	0.4–3	2–6	4/7	7/7	5/7	GTC	Diffuse white matter T2 high signal intensity with significant delay myelination for the patient's age.	5 deceased and 2 alive
		EIF2B2	2	1:1	2	3.5	8	2/2	2/2	0/2	0/2	Bilateral supratentorial confluent white matter increased signal intensity sparing the juxtracortical white matter with multiple areas of linear and dot-like white matter	Alive with severe developmental delay
		EIF2B3	2	0:2	0.6–2	3	7,9	2/2	4/4	4/4	Focal	Diffuse bilateral periventricular and deep white matter T2 high signal intensity extending into the subcortical region. It shows diffuse low signal intensity on T1 weighted	Alive with severe developmental delay
3	Peroxisome biogenesis disorders (PBD) (10)	*PEX1*	4	2:2	0–0.3	0–0.2	0.3–0.6	0/4	4/4	3/4	Focal	Focal abnormal signal intensity seen at the posterior left putamen / external capsule which shows high signal intensity on T2 /FLAIR sequence with diffusion restriction, most likely hypoxo-ischemic foci.	All deceased
		HSD17B4	3	2:1	0–0.2	0–0.6	0.6–0.8	0/3	3/3	2/3	Focal	Hyperintensity in the peritrigonal whitematter on both sides with splenium involvement of the corpus callosum	2 alive and 1 died
		PEX13	1	1:0	0	0.3	0.3	0	1	1	Focal	Peritrigonal white matter disease and thinning corpus callosum	Deceased
		PEX16	1	0:1	0	5	6	1	1	0	0	White matter disease and thinning corpus callosum	Alive with severe developmental delay
		PEX6	1	1:0	1	1.5	3.3	0	1	1	Focal	White matter disease and	Alive with severe developmental delay
4	Aicardi Goutieres syndrome (AGS) (9)	RNASEH2B	3	2:1	0–1	1–4	3–7	0/3	3/3	2/3	Myoclonic	Local areas of hyperintensity on T1W images with corresponding hypointensities on T2W images are observed bilaterally within the basal ganglia and periventricular white matter bilaterally consistent with distribution seen on CT scan suggestive of calcific foci. Preventricular leukomalacia, CNS calcification.	Alive
		RNASEH2A	2	1:1	0–1	1,2	5	0/2	2/2	0/2	0/2	Multifocal T2 high signal intensity.	Alive
		RNASEH2C	1	1:0	0.1	0.7	3	0/1	1/1	1/1	GTC	Interval progression of lost white matter bulk with atrophic changes seen in allover supra and infra tentorial structures with prominent CSF spaces and ventricles. No changes regarding focal calcification in pons with development of bilateral basal ganglia.	Alive
		IFIH1	1	0:1	0.8	3	4	1/1	1/1	0/1	0/1	Abnormal white matter distribution.	Alive
		SAMHD1	1	1:0	0	0.3	1	0/1	1/1	1/1	GTC	Multiple patchy areas of high signal intensity on T2-weighted and FLAIR sequences.	Alive
		TREX1	1	0:1	4	5	5.5	0	0/1	0	0	Abnormal diffused white matter.	Alive
5	Krabbe Disease (7)	GALC	7	4:3	0.3–4	1–5	5–12	6/6	6/6	3/6	Clonic, myoclonic	Bilateral diffuse symmetrical T2 abnormal high signal intensity involving the deep white matter with sparing of the immediate subcortical white matter. It extends from the centrum semiovale, corona radiata, periventricular white matter and involving the posterior limb of the internal capsule. Diffusion-weighted images show no evidence of diffusion restriction.	2 deceased and 4 alive
6	Pelizaeus-Merzbacher-Like Disease (6)	GJC2	6	3:3	0–0.6	0.3–3	4–10	0/6	6/6	0/6	0/6	Extensive bilateral diffuse T2 hyperintensity of the white matter with relative sparing of the subcortical white matter in the temporal lobe. Involvement of the pons and middle cerebral peduncle is noted.	Alive with severe developmental delay
7	Megalencephalic Leukoencephalopathy with subcortical cyst (MLC) (5)	MLC1	4	2:2	2–7	1–19	7–25	0/4	1/4	2/4	GTC	Bilateral symmetric abnormal white matter cerebellar and cerebellum signal on T1 and T2. Early subcortical cystic changes are seen in the anterior temporal lobes bilateral.	Alive with severe developmental delay
		HEPACAM	1	1:0	0.7	7	15	0/1	1/1	1/1	Focal	Diffuse supratentorial superficial and deep white matter disease with relative sparing of the basal ganglia. There is partial involvement of the dorsal brainstem tracts. There is involvement of the deep cerebellar white matter and dented nuclei. Small subcortical.	Alive with severe developmental delay
8	Canavan disease (4)	ASPA	4	4:0	0–1	0.5–2	2–6	1/4	4/4	1/4	GTC	Extensive diffuse bilateral low T1, high T2 white matter hyperintensity with modified restricted diffusion involving subarcuate U fibers, bilateral globi pallidi, thalami, brain stem and cerebellum is observed.	Alive
9	X-Linked Adrenoleukodystrophy (3)	ABCD1	3	3:0	4–5	5–6	7–8	3/3	3/3	3/3	Focal	Extensive symmetrical white matter changes in the parieto-occipital region on axial FLAIR sequence. Post gadolinium contrast T1 weighted images shows peripheral enhancement	All alive with severe developmental delay
10	Alexander Disease (2)	GFAP	2	1:1	0.3,2	0.4,7	0.4, 8	0/0	1/1	2/2	Focal, GTC	Bilateral diffuse symmetrical white matter signal abnormalities predominantly involving the frontal and anterior parietal lobes	Alive
11	Pelizaeus-Merzbacher disease (PMD) (2)	PLP1	2	2:0	0.5–1	1–3	5–7	0/2	2/2	0/2	0/2	Abnormal CNS demyelination	Alive with severe developmental delay
12	Salla disease (2)	SLC17A5	2	1:1	0.8–1	4–5	7–9	0/2	2/2	1/2	Myoclonic	Diffuse supratentorial abnormal signal involving the superficial and deep white matter. There is relative sparing of the cortex and deep gray matter and hypomyelination	Alive with severe developmental delay
13	Oculodentodigital dysplasia (1)	GJA1	1	0:1	0.1	6	7	0/1	1/1	0/1	0	Scattered small foci of hyperdensities in right subcortical frontal region and frontparietal region likely calcifications with high attenuation of the basal ganglia. No white matter changes noted	Alive with mild mental retardation

**Table 3:** Summary of genetic data.

**#**	**Disorders/Total**	**Gene**	**Number of patients**	**Zygosity (variant type)**	**NM number**	**Nucleotide change**	**Protein change**	**Status**	Estimated MAF (x 10^**5**^) in KSA^*^
1	Metachromatic leukodystrophy (21)	*ARSA*	9	Homozygous (splicing)	NM_001085425.3	c.1108-2A>G	p.?	Reported	30
			1	Homozygous (missense)	NM_001085425.3	c.883G>A	p.Gly295Ser	Reported	-
			1	Homozygous (missense)	NM_001085425.3	c.809T>C	p.Leu270Pro	Novel	-
		*PSAP*	10	Homozygous (missense)	NM_002778.4	c.722G>C	p.Cys241Ser	Reported	68
2	Leukoencephalopathy with vanishing white matter (VWM) (11)	*EIF2B4*	7	Homozygous (missense)	NM_001318965.1	c.1132C>T	p.Arg378Trp	Novel	15
		*EIF2B2*	2	Homozygous (missense)	NM_014239.4	c.591C>G	p.Phe197Leu	Novel	-
		*EIF2B3*	2	Homozygous (missense)	NM_020365.5	c.32G>T	p.Gly11Val	Reported	-
3	Peroxisome biogenesis disorders (PBD) (10)	*PEX1*	4	Homozygous (nonsense)	NM_000466.3	c.2176C>T	p.Gln726Ter	Reported	11
		*HSD17B4*	3	Homozygous (missense)	NM_000414.2	c.2207T>A	p.Leu736His	Reported	-
		*PEX13*	1	Homozygous (start-loss)	NM_002618.3	c.1A>G	p.Met1Val	Novel	15
		*PEX16*	1	Homozygous (splicing)	NM_057174.2,	c.113-1G>C	p.?	Novel	-
		*PEX6*	1	Homozygous (missense)	NM_000287.3	c.1931G>A	p. Arg644Gln	Novel	8
		*RNASEH2B*	3	Homozygous (missense)	NM_024570.4	c.356A>G	p.Asp119Gly	Reported	38
4	Aicardi Goutieres syndrome (AGS) (9)	*RNASEH2A*	1	Homozygous (missense)	NM_006397.3	c.202T>G	p.Ser68Ala	Novel	-
			1	Homozygous (missense)	NM_006397.3	c.557G>A	p.Arg186Gln	Reported	118
		*RNASEH2C*	1	Homozygous (missense)	NM_032193.4	c.202C>G	p.Leu68Val	Novel	4
		*IFIH1*	1	Heterozygous (missense)	NM_022168.4	c.1850T>C	p.Ile617Thr	Novel	8
		*SAMHD1*	1	Homozygous (missense)	NM_015474.4	c.428G>A	p.Arg143His	Reported	-
		*TREX1*	1	Heterozygous (missense)	NM_130384.3	c.223G>A	p.Glu75Lys	Reported	-
5	Krabbe disease (7)	*GALC*	1	Homozygous (missense)	NM_000153.4	c.916G>A	p.Ala306Thr	Reported	4
			1	Homozygous (nonsense)	NM_000153.4	c.396G>A	p.Trp132Ter	Reported	-
			1	Homozygous (missense)	NM_000153.4	c.1685T>C	p.Ile562Thr	Reported	-
			4	Homozygous (missense)	NM_001201402.1	c.1886C>G	p.Pro629Arg	Novel	-
6	Pelizaeus-Merzbacher-Like Disease (PMLD) (6)	*GJC2*	3	Homozygous (frameshift)	NM_020435.4	c.1134_1144del	p.Ala379GlyfsTer109	Reported	-
			3	Homozygous (frameshift)	NM_020435.4	c.107delT	p.Ile36ThrfsTer3	Novel	8
7	Megalencephalic Leukoencephalopathy with subcortical cyst (5)	*MLC1*	3	Homozygous (frameshift)	NM_139202.3	c.686_687delCAinsAG	p.Ser229Ter	Novel	-
			1	Homozygous (splicing)	NM_139202.3	c.177+1G>T	p.?	Reported	-
		*HEPACAM*	1	Compound heterozygous (missense and splicing)	NM_152722.5	c.416T>C, c.949-2A>G	p.Leu139Pro, p.?	Novel	-, 8
8	Canavan Disease (4)	*ASPA*	4	Homozygous (frameshift)	NM_000049.4	c.312_313delCA	p.Asp104GlufsTer2	Novel	-
9	X-Linked Adrenoleukodystrophy (3)	*ABCD1*	3	Homozygous (missense)	NM_000033.4	c.542A>G	p.Tyr181Cys	Novel	-
10	Alexandar Disease (2)	*GFAP*	1	Heterozygous (missense)	NM_002055.5	c.230A>G	p.Asn77Ser	Reported	-
			1	Heterozygous (missense)	NM_002055.5	c.715C>T	p.Arg239Cys	Reported	-
11	Pelizaeus-Merzbacher disease (PMD) (2)	*PLP1*	2	Homozygous (missense)	NM_000533.5	c.115G>A	p.Ala39Thr	Reported	-
12	Salla disease (2)	*SLC17A5*	1/2	Homozygous (missense)	NM_012434.5	c.406A>G	p.Lys136Glu	Novel	8
			1/2	Homozygous (missense)	NM_012434.5	c.116G>A	p.Arg39His	Novel	19
13	Oculodentodigital dysplasia (1)	*GJA1*	1	Heterozygous (missense)	NM_000165.4	c.125A>G	p.Glu42Gly	Novel	4

* *Based on the numbers of carriers detected among 13,180 WES/WGS performed at CENTOGENE*.

### Clinical and Molecular Landscapes of LD

**Figure 1:**
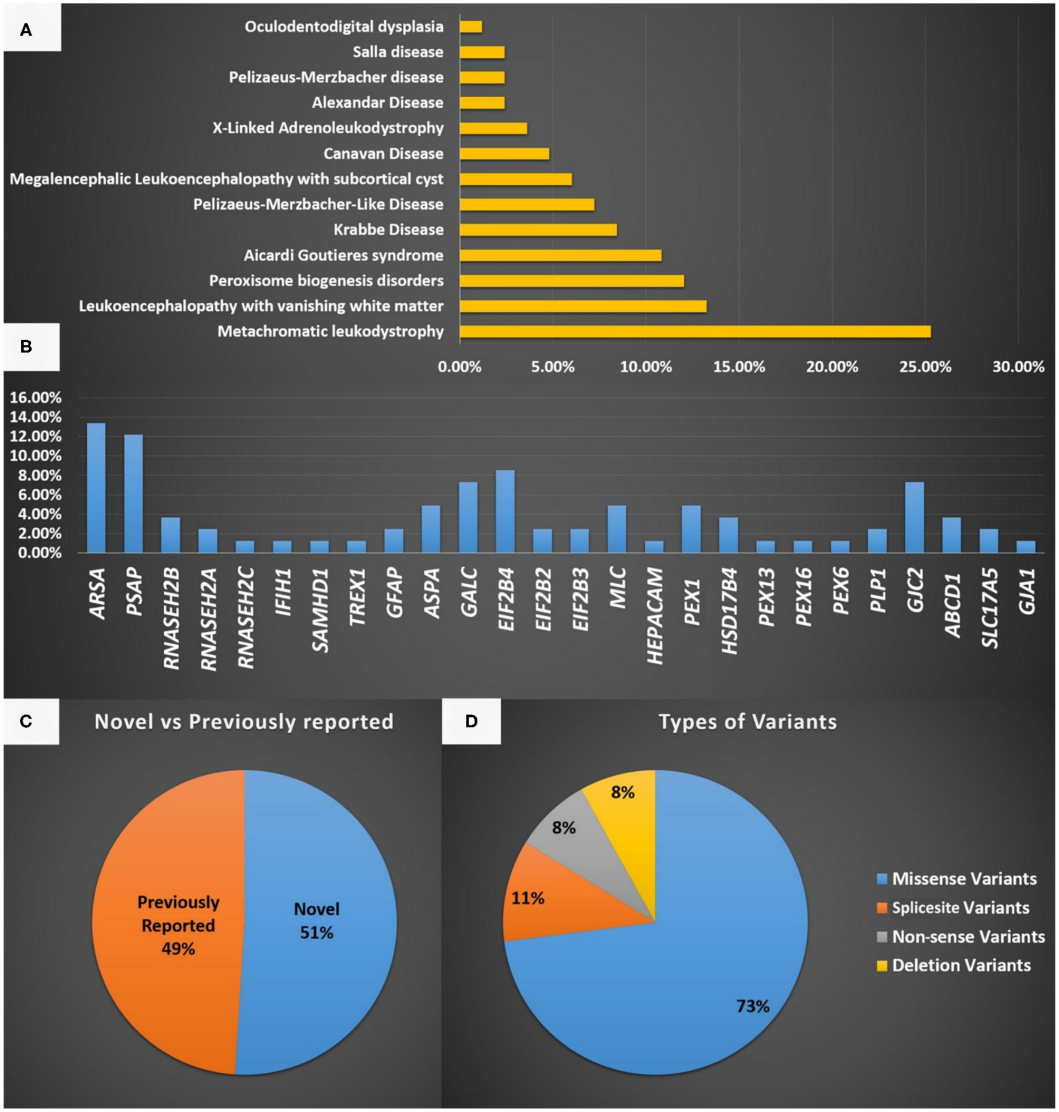
**(A)** A representation of the different LD disorders included in our cohort and their percentages. **(B)** The genes reported in the LD disorder cohort in the present study. **(C)** Representation of novel and previously reported variants identified in the present study and the type of mutations identified. **(D)** Represent the type of variants identified in the present study.

**Figure 2:**
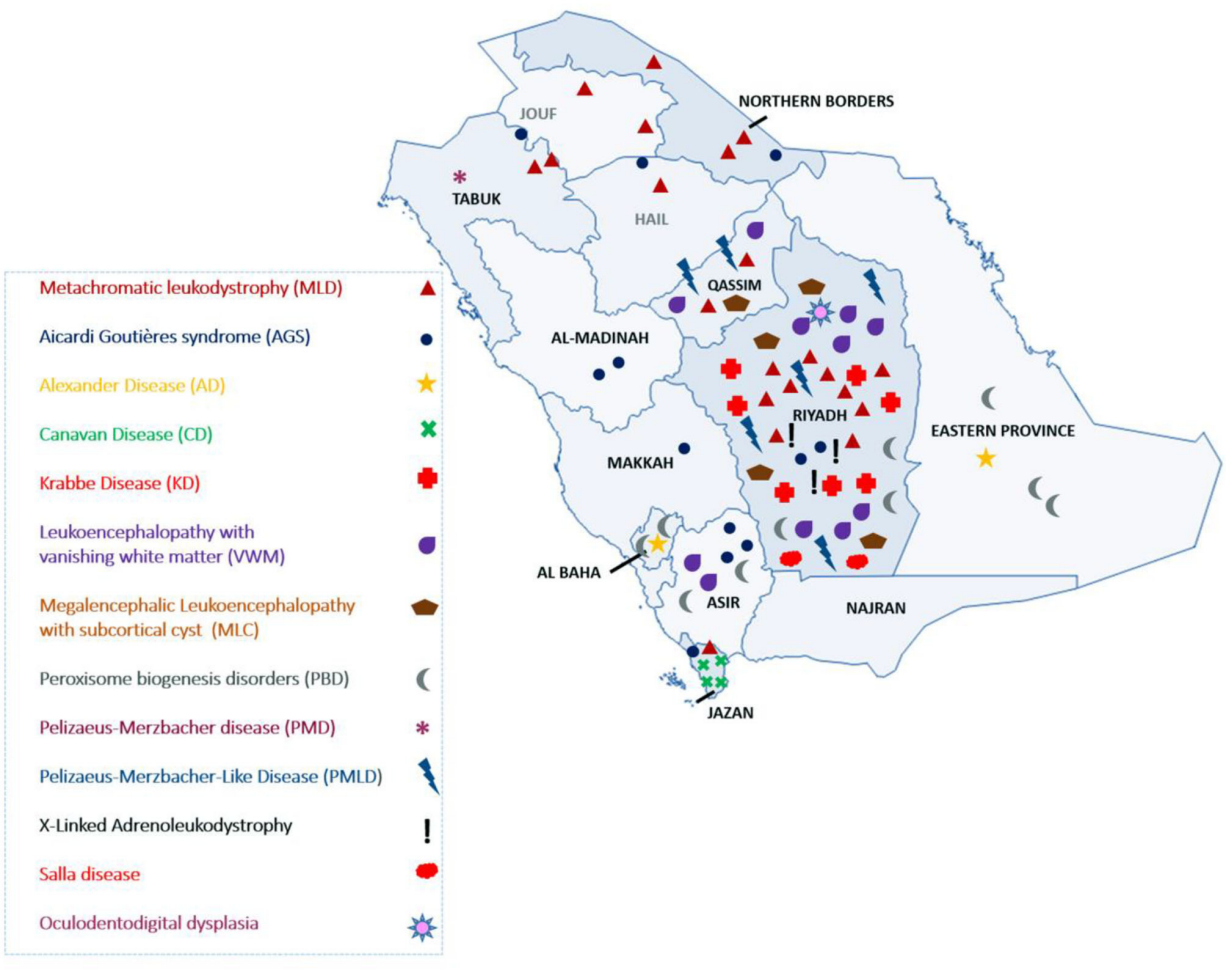
Geographical distribution of LD in Saudi Arabia.

The most common LD disorder in our cohort was MLD, which represented 25.30% of the cases. The other ordered distribution of other LD included leukoencephalopathy with vanishing white matter (VWM; 13.25%), peroxisome biogenesis disorders (PBD; 12.04%), AGS (10.84%), Krabbe disease (KD; 8.43%), Pelizaeus-Merzbacher-like disease (PMLD; 7.22%), megalencephalic leukoencephalopathy with subcortical cyst (MLC; 6.02%), Canavan disease (CD; 4.81%), X-ALD (3.61%), Alexandar disease (AD; 2.40%), Pelizaeus-Merzbacher disease (2.40%), Salla disease (SD; 2.40%), and oculodentodigital dysplasia (1.20%; Figure 1A). The central region of the country exhibited the highest prevalence of LD (48.5%). The ordered regional distribution of LD included the South Region (21%), North Region (15%), Eastern Province (10.5%), and Western regions (5%; Figure 2). However, based on our local genetic database combined with other available databases, the ordered carrier frequency of various LDs was estimated to be the highest in the Saudi population, and these LDs included AGS, MLD, leukoencephalopathy with brainstem and spinal cord involvement, and lactate elevation (LBSL, OMIM#611105), PBD, and VWM diseases (Table 2). Interestingly, polyglucosan body disease (PGBD; OMIM #263570) was present in the database but not in the clinical cohort, in which the estimated mean allele frequency of the identified pathogenic variant in *GBE1* (NM_000158.4: c.998A>T; p.[Glu333Val)] was 15 in 100,000. Similarly, LBSL with identified pathogenic variants in *DARS2* (NM_018122.4: c.1762C>G; p.[Leu588Val] was present with a mean allele frequency of 57 in 100,000.

The most common gene defects in the clinical cohort occurred in *ARSA* and *PSAP* and accounted for 13.25 and 12.04% of the cases, respectively. The most common gene for VWM was *EIF2B4*, for PBD was *PEX1*, for AGS was *RNASEH2B*, and for MLC was *MLC1* (Figure 1B).

Novel variants were discovered in 51% of the cases, whereas variants in 49% of the cases were previously reported. The most common variants were missense (73%), splice site (10.8%), non-sense (8.1%), and deletion variants (8.1%; Figures 1C,D). Most of the variants were detected in autosomal recessive genes, whereas only five variants were detected in autosomal dominant genes (13.5%). This is consistent with the high rate of consanguinity observed in our cohort (58.5%). All the identified novel variants were highly conserved across multiple species. All variants identified in the present study were uploaded to the public repository LOVD (https://www.lovd.nl/2.0/index.php).

### Radiological Spectrum of LD

**Figure 3:**
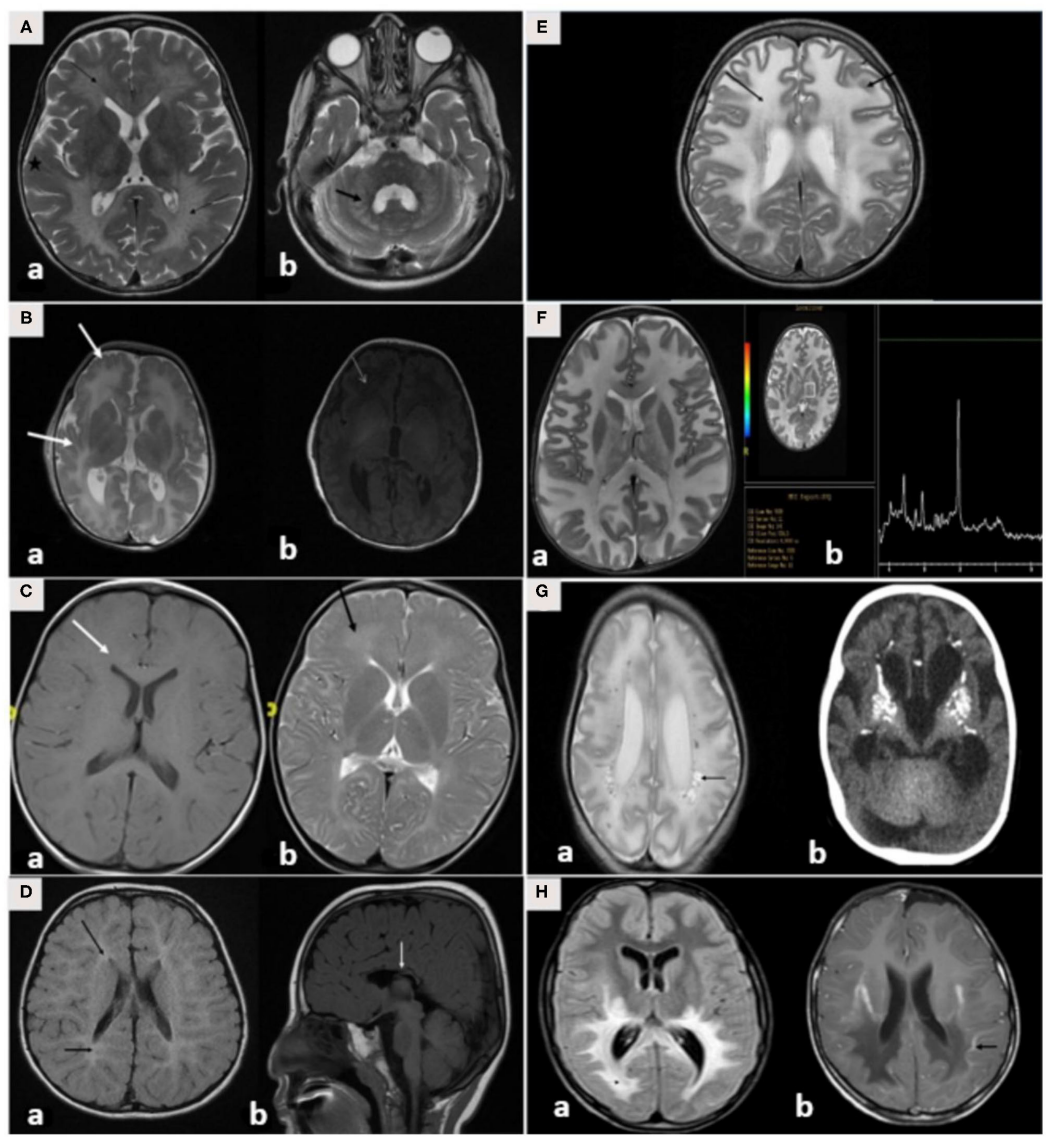
**(A–H)** MRI/CT brain of patients included in this study. **(A)** Brain MRI of a 30-month-old boy with metachromatic leukodystrophy. (a) Axial T2WI demonstrates bilateral symmetrical confluent areas of periventricular deep white matter signal change (thin arrow) with sparing of subcortical U-fibers (star). Note the classic tigroid pattern. (b) Axial T2WI exhibits bilateral involvement of the cerebellar white matter (thick arrow) and the pons. **(B)** Brain MRI of a 10-day-old boy with Zellweger syndrome. (a) Axial T2WI shows malformation of the cerebral cortex in both sylvian fissures (thick arrow) extending into the pericentral cortex and right parietal cortex as well as at the mesial aspect of the hemispheres in the form of a polymicrogyric pattern. (b) Axial T1WI shows an excessive hypointensity of the deep and subcortical white matter and also noted in the bilateral frontoparietal white matter (thin arrow). **(C)** Brain MRI of a 3-year-old boy with GJC2-hypomyelating leukodystrophy. (a) Axial T1WI shows iso/low signal intensity of the white matter (white arrow). (b) Axial T2WI exhibits an increased signal intensity of cerebral hemispheric white matter (black arrow). **(D)** Brain MRI of a 4-year-old boy with Salla disease. (a) FLAIR-WI demonstrates abnormal periventricular white matter signals (black arrow). (b) Sagittal T1WI shows thin dysplastic corpus callosum (white arrow). **(E)** Brain MRI of 11-year-old girl with megaloencephalic leukodystrophy with subcortical cysts. Axial T2WI shows diffuse superficial and deep white matter disease (thin arrow) with subcortical cysts (thick arrow). **(F)** Brain MRI of a 13-month-old boy with Canavan disease. (a) Axial T2WI demonstrates diffuse high signal intensity in the subcortical, deep, and periventricular white matter. The abnormalities also involve both globi pallidi, the thalami, and the posterior portion of the corpus callosum. (b) MR spectroscopy demonstrates increased N-acetylaspartate (NAA) peak intensity at 200 ms. **(G)** A 7-month-old-boy with Aicardi-Goutieres syndrome. (a) Brain MRI with axial T2WI at 29 days of age shows diffuse white matter with cystic changes in the periventricular areas (black arrow). (b) CT brain at the age of 6 months shows diffuse calcification in the basal ganglia. **(H)** Brain MRI of a 7-year-old boy with X-linked adrenoleukodystrophy. (a) Axial FLAIR-WI demonstrates extensive symmetrical white matter changes in the parieto-occipital region (thin arrow). (b) Gadolinium T1WI shows peripheral enhancement (thick arrow).

Brain MRI findings (Table 3; Figures 3A–H) revealed diffuse white matter disorders with demyelination in MLD, X-ALD, KD, and AD. A tigroid pattern was found in MLD and VWM disease. Periventricular predominance was observed in the MLD and KD groups. Parieto-occipital predominance was observed in X-ALD with post-gadolinium peripheral enhancement. Hypomyelination was observed in patients with SD. White matter disorders with vacuolization were found in patients with CD, VWM disease, and MLC. Brain calcification was present in the AGS and Zellweger syndrome patients. Hyperintensity in the peritrigonal white matter on both sides with splenium involvement of the corpus callosum and thinning of the corpus callosum was observed in PBD.

## Discussion

Herein, we report the largest cohort to date of LD in Saudi Arabia. Most LD cases were recruited from hospitals in the Riyadh region (Figure 2). The referral centers in this study were based in Riyadh, the capital city of Saudi Arabia. However, as inferred from their tribal origins, specific LD was prevalent in other regions. For example, CD was more prevalent in the Jazan region, PMLD was more common in the Qassim region, and MLD (due to saposin b deficiency) was more common in Dawadmi city (Figure 2).

Interestingly, the most common disorder in the clinical cohort is MLD; however, the most common disorder based on the carrier frequency of our local Saudi genome database is AGS. However, if we add the previously published data regarding AGS by Al Mutairi et al. (7) to this cohort, AGS becomes the most common LD in Saudi Arabia (8). MLD due to saposin b deficiency is more common in our population than it is in the other populations as it accounted for 12% of the total LDs.

*PSAP* defects are rare, with fewer than 30 cases reported in the literature from Italy, Turkey, India, Mexico, Canada, the Czech Republic, and Nigeria (9). Remarkably, this disease was ranked among the highest by both our clinical cohort and the local genome data, thus supporting its prevalence in Saudi Arabia. Of note, PMLD was more common than was PM. However, the prevalence of X-ALD was lower than expected in the present study.

In the present study, we estimated a minimum prevalence of 2.2/100,000 to 3.1/100,000 in Saudi Arabia. The incidence of LD is estimated to be 2.0/100,000 in Germany, 3.1/100,000 in the United Kingdom, 0.5/100,000 in France, 2.5/100,000 in the United States, and 1/100,000 in the Netherlands (9–11); however, we believe that the reported prevalence in this study is underestimated as the current study is limited to pediatric patients from hospitals located in Riyadh only.

Unsurprisingly, this study reports a high rate of novel variants (51%) as this is the first relatively large-scale report of LDs from Saudi Arabia. In correlation with the clinical, segregation, and biochemical data, the pathogenic effects of all identified novel variants were further supported by several online *in silico* prediction tools. Additionally, all of these novel variants were absent or very rare in general population databases and were not found in our in-house 2000 WES and WGS. Furthermore, all identified variants were perfectly segregated with other family members.

Naidu et al. (8) classified LD into four major categories that included hypomyelinating disorders (HMDs) that are defined as a lack of myelin accumulation, demyelinating LD that is defined by the loss of previously formed myelin, dysmyelinating disorders that possess the deposition of biochemically or structurally abnormal myelin, and myelinolytic diseases that are characterized by the vacuolization of the myelin (12). These categorical classifications of LD were based on the mechanism of white matter injury.

Subsequently, Van der Knaap and Bugiani (13) propose a classification that depends predominantly upon the primary involvement of any white matter constituent. The categories in this classification are myelin disorders due to a primary defect in oligodendrocytes or myelin (hypomyelinating and demyelinating LDs or LDs with myelin vacuolization), leuko-axonopathies, microgliopathies, leukovasculopathies, and astrocytopathies (13). Finally, Vanderver et al. (2) defined LD based on the Delphi approach, and they included 30 disorders as LDs.

We prefer to follow Vanderver's classification in the present study. This made our study more systematic and resulted in the exclusion of important diseases that were among Van der Knaap's classification (2). For example, multiple sulfatase deficiency is not included in the GLIA classification. We suggest that multiple sulfatase deficiencies should be included as a type of LD as it is a demyelinating disease. Multiple sulfatase deficiency is an autosomal recessive disorder caused by formylglycine-generating enzyme deficiency due to *SUMF1* defects. The typical clinical presentation is developmental regression, periventricular white matter disease, intellectual disability, and ichthyosis. Hijazi et al. (14) reported the presence of this disease in six children from Saudi Arabia ranging from 5 to 13 years of age, indicating that the disease is not uncommon in the Saudi population (14). Taken together, heterogeneity in the LD spectrum causes the estimation of their collective prevalence to become very difficult and to be underestimated worldwide. The clinical features and MRI findings of the current cohort were similar to those reported previously (Table 3; Figure 3).

A pattern recognition approach using brain MRI in addition to detailed history and examination would be very useful to achieve a diagnosis. Three brain MRI findings can differentiate between different types of LD. The first is the presence or absence of hypomyelination, and this is defined as no change in the MRI pattern of deficient myelination in at least two MRIs (6 months apart) in children older than 1 year. Second, to determine if the white matter defects are isolated, multifocal, or confluent. Multifocal changes indicate acquired causes, such as structural chromosomal disorders, infectious diseases, and vasculopathies, whereas confluent abnormalities indicate LD. Finally, the predominant localization of white matter abnormalities, such as parieto-occipital predominance in X-ALD, periventricular abnormalities in MLD, subcortical in CD, and diffuse cerebral in VWM diseases, must be assessed (3).

The diagnosis is achieved through a detailed clinical history, examination, and brain MRI. MRI patterns help differentiate among different types of LD. Additionally, biochemical abnormalities such as metabolites and enzyme assays are very useful tools (e.g., very long chain fatty acids for X-ALD and arylsulfatase A enzyme assays in patients with MLD) (3, 4). However, a definitive diagnosis is achieved through molecular testing and identification of pathogenic variants in the culprit genes. The advent of WES and WGS has led to an improvement in the diagnostic yield of molecular testing in LD and a significant decrease in the percentage of unsolved cases (<30%) (15). Additionally, WES and WGS provide a unique opportunity for the discovery of candidate genes such as *ISCA1* and *ISCA2* (12, 16, 17).

Treatments are typically supportive, and there are no curative treatments for LD. Patients and families require frequent counseling sessions, discussions regarding the importance of family screening, preventive measures for future pregnancies, and the establishment of multidisciplinary team care (4, 18).

Anecdotal data exist for specific diseases, but in general, this is not the case for LD. Al-Hassnan et al. (19) reported nine children from four unrelated Saudi families who were diagnosed with metachromatic LD due to sphingolipid activator protein B deficiency (19). Additionally, our group described 24 patients with AGS (7). However, the incidence, prevalence, and genetic landscape of LD in Saudi Arabia remain unknown.

Various promising therapeutic approaches currently exist in experimental settings with potential clinical translational utilities, including enzyme-replacement therapy, bone marrow transplantation, gene therapy by *ex vivo* transplantation of genetically modified hematopoietic stem cells and adeno-associated virus (AAV)-mediated gene therapy delivered to the CNS (20). Several clinical trials are currently underway that involve injecting genes via intraparenchymal, intracerebroventricular, and intracisternal routes using AAV. This method expresses the protein locally, persistently, and permanently through the use of a one-time administration (20).

## Conclusion

To the best of our knowledge, this is the largest cohort of LD from Saudi Arabia that presents epidemiological, clinical, radiological, and genetic data. Furthermore, we report 18 novel variants in 61 families that were not previously associated with LD. National and international registries are required to further delineate the epidemiological, clinical, radiological, and genetic data of the LD spectrum. Sharing the genotypic and phenotypic data of a large molecularly characterized LD cohort will improve the diagnostic rate of LD patients in the targeted population and beyond. Future multicentric studies will undoubtedly improve our understanding of the natural history of these diseases and facilitate evidence-based guidelines stipulating the disease pathogenesis that may lead to the design of new therapies and prompt treatment.

## Data Availability Statement

The datasets presented in this study can be found in online repositories. The names of the repository/repositories and accession number(s) can be found at: https://www.lovd.nl/, Individual #00358887; https://www.lovd.nl/, Individual #00335517; https://www.lovd.nl/, Individual #00334384; https://www.lovd.nl/, Individual #00335515; https://www.lovd.nl/, Individual #00335516; https://www.lovd.nl/, Individual #00335518; https://www.lovd.nl/, Individual #00358876.

## Ethics Statement

The studies involving human participants were reviewed and approved by KAIMRC. Written informed consent to participate in this study was provided by the participants' legal guardian/next of kin. Written informed consent was obtained from all participating family members (or parents in the case of minors) for publication of this research and any accompanying clinical data and images after a genetic counsellor explained the nature and possible consequences of the study to them.

## Author Contributions

MAlf: performed the majority of work associated with preparing, writing and submitting the manuscript and contributed to the clinical diagnosis and management of the patients from KASCH, KAMC. MAlma, FAM, MU, MAlg, HA, RA, FB, AAls, WE, WAlt, and AAlf: edited the manuscript, collected the data and contributed to the clinical diagnosis and management of the patients from KASCH, KAMC. MAlma, MS, AA-A, and EF: collected the data and contributed to the clinical diagnosis and management of the patients from KFMC. MAlg: collected the data and contributed to the clinical diagnosis and management of the patients from KKUH. FA and WAls: edited the manuscript, collected the data and contributed to the clinical diagnosis and management of the patients from KKUH. DB-A and AA-R: edited the manuscript, collected the data and contributed to the clinical diagnosis and management of neurological features of the patients. MB: edited the manuscript, collected the molecular genetic data. NA: collected the data and interpret WES data from KFMC. AAlh, AAls, KH, and BT: edited the manuscript collected the genetic data from Prince Sultan Military Medical City. AB-A, PB, and CB: edited the manuscript, collected the genome data from CENTOGEN, Germany. MAlmu: performed clinical evaluation of patients and collected clinical data. The final version of the manuscript read and approved by all named authors.

## Conflict of Interest

The authors declare that the research was conducted in the absence of any commercial or financial relationships that could be construed as a potential conflict of interest.

## Supplementary Material

Supplementary Figure 1 WGS/WES variant filtration steps.

## Abbreviations

LD: Leukodystrophies
MLD: Metachromatic leukodystrophy
VWM: leukoencephalopathy with vanishing white matter
PBD: Peroxisome biogenesis disorders
AGS: Aicardi Goutieres syndrome
LBSL: Leukoencephalopathy with brainstem and spinal cord involvement and lactate elevation
PMLD: Pelizaeus-Merzbacher like disease
GLIA: Global Leukodystrophy Initiative Consortium.
