# Supplementary figures and images for: The Leukodystrophy Spectrum in Saudi Arabia: Epidemiological, Clinical, Radiological, and Genetic Data
Source: Front Pediatr. 2021 May 13;9:633385. doi: 10.3389/fped.2021.633385 (PMC8155587; doi:10.3389/fped.2021.633385)

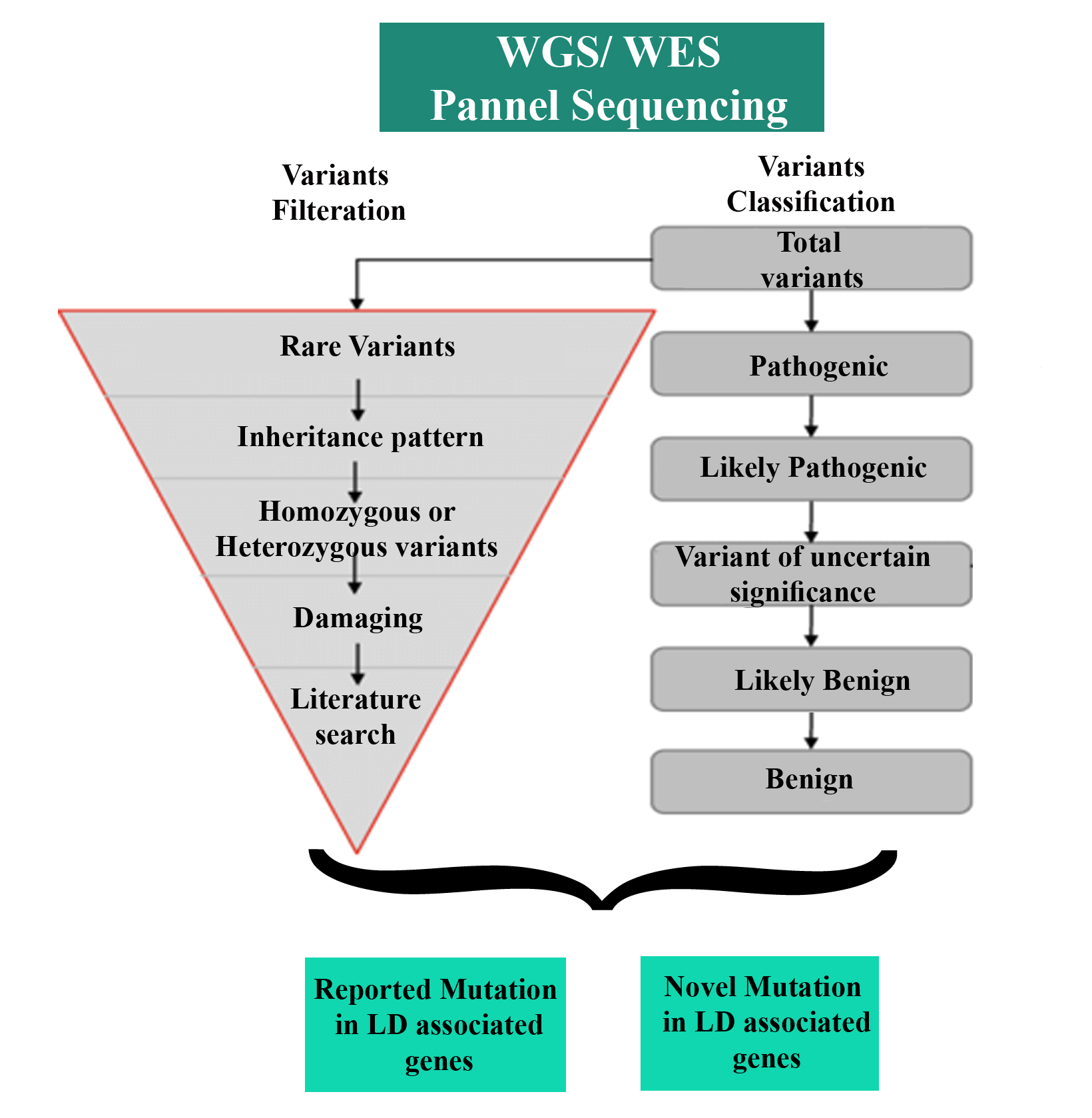

Supplement: Supplementary Figure 1 — WGS/WES variant filtration steps. [file Image_1.jpg]
